# Supplementary material for: Research on the mechanism of pore structure on water transportation in cement-based materials
Source: PLoS One. 2025 Jul 11;20(7):e0327659. doi: 10.1371/journal.pone.0327659 (PMC12250452; doi:10.1371/journal.pone.0327659)
Supplement: Attachment — Submitted filename: Responses to Reviewer s Comments-buildings-PONE-D-25-11920.doc [file pone.0327659.s003.doc]

**Responses to Reviewers’ Comments for the paper entitled**

**Study on chloride attack resistance of concrete with lithium slag content**

Buildings

Manuscript ID: buildings-PONE-D-25-11920

by Enze HAO，Yuhang LI,Dali ZHANG，Wenbang ZHU，Ruiming LIU，Xinjie WANG，Yali CAO,Yali GU, Xiumei ZHENG

The authors wish to thank the editor and five reviewers for their very thorough, insightful, and constructive reviews. All comments have been addressed in the revised version of the manuscript. Original comments of each reviewer have been placed in boxes in this document and specific responses have been placed in italics for ease of reference. In the responses, all the numbers of figures, tables, and lines refer to the revised manuscript, unless otherwise specified.

**Reviewer #1:**

*The authors thank the reviewer for the valuable and careful comments. All of the following comments have been addressed in the revised manuscript.*

*In order to improve the quality of this paper, the full test has been checked and corrected in the revised manuscript.*

1 Although both MIP and NMR techniques were used for porosity characterization, the paper fails to rigorously reconcile the different pore size classification schemes inherent to these methods. The thresholds defining “harmless,” “slightly harmful,” and “harmful” pores are applied equally to both NMR and MIP outputs, despite their differing sensitivities and principles. A critical comparative calibration is needed, particularly when drawing mechanistic conclusions based on porosity proportions.

*The authors are grateful to the reviewer's precious comment.In this study, the pores are classified into four categories following the classification proposed by Academician Wu Zhongwei. The first category is harmless pores, with diameters ranging from 0 nm to 20 nm. These pores are usually closed or isolated and do not form a connected network. They hardly reduce the mechanical strength of the material and have no significant contribution to permeability. The second category is slightly harmful pores, with diameters ranging from 20 nm to 50 nm. These pores may form local connected paths, but the overall permeability is relatively low. They slightly weaken the strength and allow a small amount of water or ions to slowly penetrate, but the threat to durability is limited. The third category is harmful pores, with diameters ranging from 50 nm to 200 nm. These pores are prone to form continuous channels, significantly increasing the material's permeability, obviously reducing the density and mechanical strength, and accelerating the invasion of harmful substances such as chloride ions and sulfates. The fourth category is highly harmful pores, with diameters greater than 200 nm. These are large pores or crack-level pores, usually caused by construction defects or shrinkage stress. They severely damage the material's strength and impermeability, becoming stress concentration points and crack propagation paths, and significantly shorten the service life of the structure. The purpose of using MIP and NMR to test the pores and adopting the same pore size distribution is to control the variables and better compare the MIP test with the NMR test. Lines 241-259 and 350-365 of the article.*

2 Figure 9 and the accompanying text suggest that an increase in water-cement ratio leads to a “more compact” pore structure based on the decreasing hysteresis loop area. This interpretation contradicts classical understanding, where increased w/c typically produces more porous structures. The decrease in hysteresis area could instead be a result of increased pore connectivity and larger average pore sizes, reducing capillary retention during desorption. This alternative explanation is not considered.

*Thanks to the reviewer for raising a very important problem.It has been considered that due to the increase in pore connectivity and the enlargement of the average pore diameter, the capillary retention during the desorption process can be reduced. This is discussed in lines 524 to 527 of the article.*

3 The SEM images presented in Figure 6 are used qualitatively to support trends observed in other analyses, but no image processing or quantification is provided. As a result, the SEM section adds limited scientific value and cannot substantiate microstructural claims quantitatively.

*The authors are grateful to the reviewer's precious comment.The SEM image in Figure 6 was processed, and this was done in lines 387 to 394 of the article.*

4 While the authors attempt to validate their COMSOL simulation model using water absorption data (Section 4), the agreement is shown only qualitatively via contour maps. No statistical or graphical comparison between simulation outputs and experimental absorption curves is offered. This limits the rigor and usefulness of the modeling section.

*Thanks. In Section 4.2 of the article, a comparison analysis between experiments and simulations has been added, starting from line 572 to line 591.*

5 The correlation between porosity (as measured by NMR or MIP) and capillary water absorption is repeatedly emphasized, yet no regression analysis or fitting (e.g., linear, power-law) is provided to quantify these relationships. This omission weakens the claim that porosity directly governs water transport dynamics.

*Thanks to the reviewer for raising a very important problem. To enhance the relationship between porosity and water absorption, a fitting graph of porosity and water absorption for MIP and NMR tests was added in the 452 to 466 lines of the article.*

6 While the T2 relaxation times are reported, there is no conversion to pore size distribution using an appropriate model. Furthermore, the authors do not distinguish between bound water and free water peaks, which are critical for understanding transport and retention mechanisms.

*Thanks for the reviewer's question. In the numerical simulation of this study, only porosity was considered, while the differences in pore distribution, free water and bound water transmission were not taken into account. There was indeed a certain error when comparing the simulation results with the experiments, but the error was within a controllable range and the overall trend was consistent. Thank you for the expert's opinion. We will make improvements in the later research.In lines 572 to 591 of the article.*

7 From MIP, porosity increases with higher w/c ratios, but the proportion of highly harmful pores in paste is lower than in mortar and concrete despite having higher total porosity. This indicates inconsistency or misinterpretation in assigning pore categories. A clearer explanation or cross-validation between MIP and NMR data is needed.

*Thanks to the reviewer for raising a very important problem.A comparative analysis of nuclear magnetic resonance and pressure generation has been added, starting from line 350 to line 365 of the article.*

8 Given the scope and content of this paper, it may benefit from considering the following related works:
https://doi.org/10.1007/978-3-031-69626-8_32
https://doi.org/10.1061/(ASCE)MT.1943-5533.0004528
https://doi.org/10.1016/j.matpr.2023.04.103

*The authors are grateful to the reviewer's precious comment.This study has referred to the above three articles and added citations, which can be found in lines 672-686 of the article.*

**Reviewer #2:**

*The authors thank the reviewer for the valuable and careful comments. All of the following comments have been addressed in the revised manuscript.*

*In order to improve the quality of this paper, the full test has been checked and corrected in the revised manuscript.*

1.It is necessary to clarify what method was used to determine the composition of the cement presented in Table 1

*Thanks for pointing out. It has been stated in lines 114-115 of the article what method was used to determine the composition of the cement.*

2. Figure 1 and the data presented in it do not carry a deep scientific load and the authors are advised to transfer this figure to the supporting information file. Similarly, Figures 2 and 11.

*Thanks for the kind remind from the reviewer. Figure 1 shows the sample fabrication and testing process, while Figure 2 presents the Microscopic instruments and test samples. The contents of Figure 1 and Figure 2 have been simplified, some pictures have been deleted, and some pictures have been placed in the attachment. Figure 12: Water absorption simulation of P, M and C specimens at different times. In Section 4.2 of the article, an experimental and simulation comparison analysis has been added, and Figure 12 has enhanced the correlation between the experiment and the simulation. This is in lines 169-174, 202-208, and563-571 of the article.*

3.Why are the data in Table 3 presented without statistical processing?

*Many thanks for the precious comment. Table 3 Humidity corresponding to saturated salt solution, Refer to [42] Hygrothermal performance of building materials and products - Determination of moisture content by drying at elevated temperature; Amendment 1: ISO 12570 AMD 1-2013 [S], 2013. (Translated from Chinese) Data obtained from the literature.This is in lines 175 and 773-775 of the article.*

4.The size scale in Figure 6 is unreadable, it needs to be improved.

*Thanks for the reviewer's question. The SEM images of cement-based materials with different water-cement ratios in Figure 6 have been replaced. Higher-precision images have been used, and the size and proportion are clearly visible. These images can be found in lines 387-395 of the article.*

5.The conclusion also looks like the conclusion of a technical report and not a research manuscript. It needs to be carefully revised

*Thanks for the reviewer's question. The conclusion of the article has been revised and can be found on lines 596 to 629.*

**Reviewer #3:**

*The authors thank the reviewer for the valuable and careful comments. All of the following comments have been addressed in the revised manuscript.*

*In order to improve the quality of this paper, the full test has been checked and corrected in the revised manuscript.*

1.lines 107-109: since for non-Chinese researchers can be difficult to find these Codes, can you provide a reference?

*Thanks for the reviewer's question.Reference materials have been provided, and they can be found on pages 769 to 772 of the article.*

2.*line 117: in fact, figure 1d shows the cutting machine, not the samples.*

*Thanks for the reviewer's question. The text has been revised. It has been changed at line 137 of the article.*

3.*line 122: can the authors explain the procedure to dry the sample? Did they use the oven at 80°C as before?*

*Many thanks for the precious comment.The water absorption experiment reached the specified time points, with durations of 5 minutes, 10 minutes, 20 minutes, 30 minutes, 1 hour, 2 hours, 3 hours, 4 hours, 5 hours, 6 hours, 10 hours, 14 hours, and 24 hours respectively. After drying the surface moisture of the specimens, the weights were measured. The weights were measured without using a drying oven. In lines 141-145 of the article.*

4.*line 130: ‘…according to the standards formulated by the National Technical Committee of Physical Stoichiometry.’ Please, give a reference.*

*Thanks for the kind remind from the reviewer.References are provided on lines 150-151 of the article.*

5.*line 144: ‘m denotes the weight…’: m denotes the mass, not the weight.*

*Thanks for pointing out.The modification is made. "m" represents mass, and it is located in line 176 of the article.*

6.*line 168: please, specify the model of SEM used.*

*Thanks.The manufacturers and models of SEM that have been added are listed in lines 177-179 of the article.*

7.*line 170 and following: how was the pressurization achieved? At a constant rate or with other means (e.g. dynamic response)?*

*Thanks.This study employed the AutoPore IV 9500 pore measurement instrument produced by the American company Micromeritics. The pore measurements within a specific pore size range were achieved through staged pressure application (low-pressure pneumatic + high-pressure hydraulic). It is necessary to strictly control the pressure gradient, equilibrium time, and safety precautions. This is stated in lines 188-189 of the article.*

8. *line 177: the SEM and the test sample…: the MIP, not the SEM*

*Thanks for the reviewer's question.This sentence has been deleted.*

9.*line 185: ‘The pore size measurement range was 0.00002 to 200 μm’: please, control the range: are you able to measure the pore size within 0.02 nm? The Bohr diameter of the hydrogen atom is 0.1 nm.*

*Thanks.The aperture measurement range has been revised to 0.0002 - 200 μm, as stated on line 201 of the article.*

10. *line 185: ‘the SEM and the test sample…: the NMR, not the SEM.*

*Thanks for the reviewer's question.This sentence has been deleted.*

11. *lines 195 and 372: analysis, not analize.*

*Thanks.The text has been revised, at lines 211 and 366 of the article.*

12. line 205: in figure 3a, b and c, ‘the volume of mercury intrusion is greater than that of mercury extrusion.’ It seems the contrary: the intrusion lines are always below those of extrusion lines.

*Thanks for pointing out.The modifications have been made in lines 221-222 of the article.*

13. *lines 206-208: ‘trend of cumulative mercury intrusion volume reduction is P1 > P2 > P3, M1 > M2 > M3, and C1 > C2 > C3, while the cumulative mercury extrusion volume undergoes negligible change.’ Concerning intrusion, P1 < P2 < P3; furthermore, extrusion volumes are significantly different for P and C samples. Only for M samples they are very similar.*

*Many thanks for the precious comment.During the drawing process of the Mercury curve of P, an error occurred and the line types of P1 and P3 were reversed. When drawing the mercury curve of C, due to the addition of coarse aggregates in the concrete, it had a certain impact on the connectivity of the concrete interior, resulting in some errors in the mercury insertion and withdrawal tests of C2 and C3 specimens. However, the overall trend was generally in line with the pattern. At line 221-227 and 276 of the article.*

14. *line 234 and following: the notion of harmless and harmful pores (in relation to durability?) should be defined.*

*Thanks for the reviewer's question.The definitions of harmless holes, slightly harmful holes, harmful holes, and highly harmful holes have been added. They are located in lines 241 to 259 of the article.*

15. *lines 238 to 250: all the quoted numbers are in my opinion useless, since the proportions are already visible in figure j and k.*

*Thanks for the reviewer's question.The description in the original text has been deleted.*

16. *line 290: 90dP: maybe 90 days for C etc.?*

*Thanks.For the specimens of mortar, concrete and concrete mixtures, they were all cured for 90 days. This was done to allow the cement to fully hydrate, prevent errors caused by the cement hydration process, and to control the variables.*

17. *line 296: apparently, a second peak is not visible from figure 4a in the range 6-3000 ms. Can you show it in an inset?*

*Thanks for the reviewer's question.In Figure 4(a), there is only one main peak, and an illustration has been added, on line 320 of the article.*

18. *line 299: ‘indicating that the larger the water-cement ratio, the more the pore size.’ Unclear statement: the larger the size? The higher the number of pores?*

*Thanks.The changes have been made in lines 302-304 of the article.*

19. *lines 328-348: as noted for MPI (lines 238-250), it is useless to repeat the numbers already shown in figure 5. It makes the text rather confuse.*

*Thanks.The description in the original text has been deleted and modified. These changes have been made in lines 324-346 of the article.*

20. *line 414: ‘…saturation at 12000’: 12000 g/m^2^. Please, specify the units.*

*Thanks.The unit "g/m2" has been added in pages 416-419 of the article.*

21. *line 415: ‘and that of P3 at 18,000.’ In fact, from the figure it seems that both P2 and P3 saturate at almost 16000 g/m^2^.*

*Thanks for the kind remind from the reviewer.The modifications have been made in lines 416-417 of the article. P2 and P3 reached saturation at approximately 15700 g/m^2^ and 16300 g/m^2^ respectively.*

22. *line 417-418: ‘The water absorption of the specimens shows that the paste is greater than the mortar.’ Greater in what sense? Please, clarify the sentence.*

*Thanks for pointing out.The modifications and additions have been made in lines 419-430 of the article.*

23. *Figure 9: can the authors add the error bars? Particularly for C samples it should be useful, in order to support the claim that the area of hysteresis loop decreases by increasing the water-to-cement ratio.*

*Thanks for the reviewer's question.In Figure 9, the area of the hysteresis loop of the isothermal adsorption-desorption curves of cement-based materials with different water-cement ratios has added error bars, as shown in line 520 of the article.*

24. *line 546: ‘When the water absorption time reaches 10 days…’ This is not shown in the figure.*

*Thanks for the reviewer's question.The description that was deleted 10 days ago has been removed.*

**Reviewer #4:**

*The authors thank the reviewer for the valuable and careful comments. All of the following comments have been addressed in the revised manuscript.*

*In order to improve the quality of this paper, the full test has been checked and corrected in the revised manuscript.*

1.Sections 3.1–3.3 should be consolidated into a single subsection with side-by-side comparisons and a schematic diagram of pore evolution to enhance readability and synthesis.

*Thanks for the reviewer's question.The sections 3.1-3.3 have been combined into 3.1 - Microscopic Analysis. A comparative analysis between MIP and NMR has been conducted, and the modifications have been made in pages 210-211, 290, 355-365, and 366 of the article.*

2.The lengthy descriptive passages (e.g., Lines 232–268, 290–320, 325–368, 412–440) should be streamlined. Some observations could be replaced with schematics or summarized more concisely.

*Thanks for pointing out.Some content has been deleted, specifically in lines 239-275，305-319，324-346，405-448 of the article.*

3.Avoid redundant subsections like "Analysis of the reasons" (Lines 258, 313)—integrate explanations directly into results.

*Thanks.The redundant sections have been deleted and the explanations have been incorporated into the results.*

4.The CFD simulation setup is not described in the Methods section, leaving readers unprepared for Section 4. Provide details on boundary conditions, meshing, and validation.

*Thanks for the kind remind from the reviewer.The information regarding the boundary conditions and grid division of the model is described in lines 539-543，549-553 of the article.*

5. Equation (2) (Line 407) lacks derivation or citation. Justify its reasonable use and include a derivation in the Supporting Material.

*Many thanks for the precious comment.Formula 2 has been cited in the references, appearing on pages 401 and 785-787 of the article.*

6.Clarify the definition of A (water absorption surface area)—is this cross-sectional area or pore surface area? (Line 408)

*Thanks.The modification has been made. A cross-sectional area of the specimen's water absorption surface, as stated in line 402 of the article.*

7. Figure 3
7.1) It is hard to read the graphs due to inconsistent color and symbols. Let me pick P1 as an example for explanation. The intrusion-extrusion curves of P1 (Figure 3(a)) should be with the same color (black) and differentiate intrusion-extrusion with symbols/line styles. Also, use the same color for P1/M1/C1.
7.2) Figures 3(g-i): Clarify the y-axis unit for cumulative mercury volume.
7.3) Figures 3(j-k): What is the number in x-axis? Is it a sample code?
7.4) Check the consistency of figure information.

*Thanks for pointing out.The colors and symbols in Figures 3(a)(b)(c)(d)(e)(f)(g)(h)(i) have been modified and unified. The y-axis unit of cumulative mercury volume in Figures 3(g-i) has been revised. In Figures 3(j-k), P1, P2, P3, M1, M2, M3, C1, C2, and C3 on the x-axis represent the specimen numbers with different water-cement ratios. This is described in lines 276-289 of the article.*

8. Lines 206-208: The authors should re-examine on the ranking of cumulative mercury intrusion volume and the explanation of cumulative mercury extrusion volume.

*Thanks.The modifications have been made in lines 221 to 227 of the article.*

9.Line 105: The abbreviation of paste, mortar, and concrete should be specified here.

*Thanks for the kind remind from the reviewer.The abbreviations for mortar, cement mortar and concrete have been added in line 124 of the article.*

10. Table 1: Is chemical composition of cement in mass fraction or mass percentage?

*Thanks for the reviewer's question.Table 1: The chemical composition of cement is expressed as mass percentages, on line 122 of the article.*

11. Table 2: Capitalize “Superplasticizer”.

*Thanks for pointing out.The changes have been made in Table 2 of the article.*

12. Line 130: Cite the source for the National Technical Committee of Physical Stoichiometry.

*Thanks.References have been added in lines 150-151、773-775 of the article.*

13. Lines 168-169, 177-178, 185-186: Please check the description.

*Thanks.The original description has been deleted at the corresponding position.*

14. Line 195: MIP analyze or MIP analysis?
Line 372: SEM analyze or SEM analysis?

*Thanks for the reviewer's question.The 211、366 line of the article has been revised.*

15. Lines 216-220: The interpretation of mercury intrusion-extrusion behavior requires clarification. The claim that “The insignificant change in the cumulative mercury volume” contradicts the visible hysteresis in Figure 3. While the extrusion curve does not mirror the intrusion trend, the disparity itself is significant and should be discussed as evidence of pore entrapment or ink-bottle effects. Moreover, the assertion that pore connectivity is "stable" (i.e., non-deformable) lacks justification. MIP hysteresis alone cannot confirm this—connectivity could evolve due to mechanical deformation or expansion of pore.

*Thanks for pointing out.This part has been deleted.*

16. Figure 5: number?

*Thanks for the kind remind from the reviewer.The X-axis number in Figure 5 has been deleted at line 347 of the article.*

17. Lines 414-417: Unit of water absorption capacity?

*Many thanks for the reviewer's suggestion.The unit of water absorption, g/m ², has been added in lines 416-419 of the article.*

18. Figure 7: Can we plot the graphs with x-axis of time (h)? I do not see the specific reason in the discussion why to select the square root of time in x-axis.

*Thanks to the reviewer for raising a very important problem.The graph can be plotted with the x-axis representing time (h), and the overall shape is a curve with weak regularity. To make the relationship between water absorption and time more obvious, especially in the early stage where it is approximately proportional, referring to Kumar et al., the water absorption per unit area is proportional to the square root of time. Therefore, the square root of time is chosen as the x-axis. This is described in lines 407-413 of the article.*

19. Figure 8: The authors have plotted the equilibrium absorption-desorption isotherms of water in cement-based materials. However, in determining the area of the hysteresis loop, increasing the number of data points for the hysteresis loop may improve accuracy. Moreover, the lower closure point of the hysteresis loop should not artificially be zero in typical. Therefore, I suggest that the authors add a greater number of points. Also, the area of the hysteresis loop can be better computed through numerical integration to reduce numerical truncation errors rather than the curve fitting with simple cubic equation at absorption and desorption branches.

*The authors are grateful to the reviewer's precious comment.We sincerely appreciate the reviewers' valuable suggestions for improving the accuracy of the analysis of the hysteresis loops in the water adsorption-desorption isotherms of cement-based materials. The key points regarding data point density, treatment of closure points, and numerical integration are highly valuable for refining our approach. Although a higher data point density theoretically can enhance precision, the current intervals are constrained by the equilibration time required for each humidity step. To balance experimental feasibility and accuracy, we prioritize the critical humidity regions where pore filling is most significant.*

*We agree that adding more data points within the adsorption-desorption curves will further enhance the reliability of the area calculation. In future studies, we will optimize the humidity intervals and employ an automated adsorption system to minimize errors.*

*In the analysis, the humidity points of the adsorption-desorption curves are not artificially set to zero. They are determined by the equilibrium moisture content measured at 5% relative humidity, which represents the residual moisture in the pores of cement-based materials under nearly dry conditions. This approach follows the established conventions for cement-based materials. While numerical integration can reduce truncation errors, fitting with a cubic polynomial can also reduce errors and thereby minimize the area error.*

20. *The first conclusion (Section 5) is overly broad and states a well-known fact. Focus on specific findings from this study*

*Thanks for the precious comment.The conclusion of the article has been revised and is on lines 596-629 of the article.*

**Reviewer #5:**

*The authors thank the reviewer for the valuable and careful comments. All of the following comments have been addressed in the revised manuscript.*

*In order to improve the quality of this paper, the full test has been checked and corrected in the revised manuscript.*

1.1. (See 2.2) 2.1. Raw Materials ---> 2.1. Raw materials
2. 2.3 Test Methodology ---> 2.3 Test methodology
3. Tab. 2. Superplasticizer ---> Superplasticizer
4. Line 144. Where ---> where
5. Tab. 3. humidness ---> Humidness
6. 5.conclusion ---> 5. Conclusion

*Thanks for pointing out. The sections 112，130-131，166，175 and 592 of the article have been revised.*

2. Fig. 3, panels (a) – (c) Why were lower pressure measurements not performed for some samples?

*Thanks for the kind remind from the reviewer. The main focus of this study is on pores with a diameter of 200 nm or less. The high-pressure section of the mercury intrusion method is sufficient to cover this range.*

3.The authors were interested in the range of pores up to 200 nm. Why did the authors not perform low-temperature measurements of nitrogen adsorption? Mercury porosimetry is a method that is burdened with error. A combination of these two methods would be very desirable.

*Many thanks for the precious comment. The nitrogen adsorption method is typically suitable for characterizing micropores (<2nm) and mesopores (2-50nm), but its measurement accuracy significantly decreases for macropores (>50nm), especially for pores close to 200nm. Due to the weak capillary condensation of nitrogen in larger pores, the data reliability is reduced, making it difficult to accurately characterize the pore distribution within the 50-200nm range. In contrast, mercury intrusion porosimetry (MIP) has a broader measurement range (typically covering 3nm - 400μm) and is particularly adept at characterizing the pore structure from mesopores to macropores (50-200 nm), fully covering the research interval of interest to the authors (the entire range below 200nm). Additionally, nitrogen adsorption requires degassing of the sample in a high-temperature vacuum environment, which may damage the structure of some sensitive materials (such as samples containing water or organic matter). In contrast, mercury intrusion porosimetry and low-field NMR are less destructive to the sample, with low-field NMR being capable of non-destructive testing, making them suitable for subsequent studies that require the preservation of the sample's original state. Therefore, this study adopts a combined approach of mercury intrusion porosimetry and low-field NMR.*

4.All figures - axis descriptions. Sometimes titles start with a lowercase letter, and sometimes with an uppercase letter. Please standardize.

*Thanks for the reviewer's question. The capitalization of the titles in the article has been corrected.*

5.Figs. 7 and 8. After the adsorption/desorption cycle, was another cycle of measurements performed on the same sample?

*Thanks for the reviewer's question. After the adsorption and desorption experiments were completed, no additional tests were conducted on the same samples. During the tests, three specimens were taken from each group. If the error was less than 10%, the average value was taken; if the error was greater than 10%, the data exceeding 10% were excluded, and the remaining values were averaged.*

6.Tab. 4. Too high precision of the given values.

*Many thanks for the precious comment.The data in Table 4 has been rounded to three decimal places, and it appears in line 517 of the article.*
